# A Pilot, Open-Label, Proof-of-Concept Study To Evaluate the Efficacy and Safety of Asthiposhak® Tablets in Participants Suffering From Asthikshaya or Osteopenia

**DOI:** 10.7759/cureus.41862

**Published:** 2023-07-14

**Authors:** Khushal Kumar, Pawankumar Godatwar, Sanjeev Sharma, Sangam Narvekar, Megha Nalawade, Mukesh B Chawda, Pragya Verma, Rajmohan Seetharaman, Raakhi K Tripathi

**Affiliations:** 1 Pathology and Diagnostic Procedures (Roga Nidana Evum Vikriti Vigyana), National Institute of Ayurveda, Jaipur, IND; 2 Pathology and Diagnostic ProceduresPathology and Diagnostic Procedures (Roga Nidana Evum Vikriti Vigyana), National Institute of Ayurveda, Jaipur, IND; 3 Surgery (Shalya Tantra), National Institute of Ayurveda, Jaipur, IND; 4 Medical Services, Shree Dhootapapeshwar Limited, Mumbai, IND; 5 Clinical Research, Shree Dhootapapeshwar Limited, Mumbai, IND; 6 Pharmacology and Therapeutics, Seth G.S. (Gordhandas Sunderdas) Medical College and KEM (King Edward Memorial) Hospital, Mumbai, IND

**Keywords:** asthikshaya, ayurvedic symptom score, fracture risk reduction, dexa scan, bone mineral density, traditional medicine, osteoporosis

## Abstract

Introduction

Both osteoporosis and osteopenia are prevalent public health concerns worldwide and can lead to debilitating bone fractures. This study aimed to assess the efficacy of Asthiposhak® Tablets in individuals with Asthikshaya (osteopenia) by measuring changes in the bone mineral density (BMD) score before and after the intervention, specifically between visit 1 (baseline) and visit 8 (after 180 days of treatment).

Methods

The single-arm study involved the screening of participants for Asthikshaya (osteopenia) using baseline investigations, which included a bone mineral density (BMD) assessment through a dual-energy X-ray absorptiometry (DEXA) scan. A total of 36 participants were enrolled in the study, who took two Asthiposhak Tablets three times a day with lukewarm water, for a period of 180 days. Safety assessments, along with evaluations of BMD (DEXA Scan), Ayurvedic Symptom Score, and serum biochemical markers, were conducted through blood investigations. Efficacy and safety data were analyzed using 'intention-to-treat' analysis. Descriptive statistics were used to express data in percentages, mean ± SD, or median (IQR). Data at different intervals were compared using paired t-tests or Wilcoxon signed-rank tests. One-way analysis of variance (ANOVA) with Bonferroni correction tested the significance between visits for the Ayurvedic Symptom Score, and Friedman's two-way analysis of variance by ranks measured differences in vital parameters. The significance level used was p<0.05.

Results

Out of the initially recruited 36 participants, 30 successfully completed the study, consisting of 12 males and 18 females, with an age range of 40 to 70 years and a mean age of 51.33 years. After 180 days of treatment with Asthiposhak Tablets, a statistically significant (p<0.05) improvement in hip and spine BMD (T-score) was observed. Additionally, significant reductions in the mean Total Ayurvedic Symptom Score were noted at both 90 and 180 days of treatment compared to day 0. Moreover, the levels of bone-specific alkaline phosphatase and osteocalcin, serum bone markers, showed statistically significant (p<0.05) reduction after 180 days of treatment compared to day 0. Importantly, all safety variables, including laboratory investigations, remained within the normal range following the 180-day treatment with Asthiposhak Tablets.

Conclusion

Asthiposhak Tablets exhibited significant efficacy in enhancing both BMD (T-score) and Ayurvedic Symptom Score, thereby substantiating their osteoprotective potential in individuals with Asthikshaya (osteopenia). Furthermore, the tablets were found to reduce the levels of biochemical markers, such as serum bone-specific alkaline phosphatase and osteocalcin, suggesting their anti-resorptive action.

## Introduction

Asthikshaya can be associated with osteopenia, which is a condition characterized by low bone mineral density that is below normal but not as severe as osteoporosis. It is often considered a precursor to osteoporosis and indicates a reduction in bone strength, making individuals with osteopenia more susceptible to fractures. Osteopenia typically has no symptoms and often remains undetected until it results in a low-impact fracture of the hip, spine, proximal humerus, pelvis, or wrist, often requiring hospitalization [1]. It affects approximately one in three women and one in 12 men, leading to significant illness, increased mortality rates, and substantial healthcare and social service costs [2]. The increased prevalence in women is because estrogen plays a crucial role in maintaining the bone health of women [3,4]. A recent study revealed that 56% of Indian men over the age of 50 and 33% of postmenopausal Indian women had osteopenia, while 7.2% of men and 5% of women had osteoporosis [5].

Dual-energy X-ray absorptiometry (DEXA) is considered the standard method for measuring bone mineral density (BMD) and is often referred to as the 'gold standard.' It provides valuable information about the likelihood of fractures caused by osteoporosis and helps in monitoring the effectiveness of treatment [6]. The management of osteoporosis aims to prevent fractures by enhancing bone strength, reducing the risk of falls and injuries, alleviating symptoms and deformities resulting from fractures, and maintaining normal physical function. Adequate intake of calcium and Vitamin D is essential for this purpose. Various medications, such as bisphosphonates, hormone replacement therapy, estrogen agonists, calcitonin, parathyroid hormone (PTH), and denosumab, are utilized based on their affordability and availability. However, it is important to note that these drugs may also be associated with adverse effects [1,7].

Ayurveda recommends the use of various herbs and minerals, either individually or in combination, for managing Asthikshaya (osteopenia) to achieve a synergistic therapeutic effect. Asthiposhak® Tablets, for instance, exert beneficial effects on bone health due to the combined actions of its ingredients [8]. Kukkutandatvak Bhasma (processed hen eggshell) provides a natural and bioavailable source of calcium, which is an essential nutrient for bone health. Shodhit Laksha (processed *Laccifer lacca*), Asthisamhruta (*Cissus quadrangularis*), Arjuna (*Terminalia arjuna*), and Babboola (*Acacia arabica*) are commonly used ingredients that help accelerate the healing of fractures [8-12]. Shodhit Guggul (processed *Commiphora wightii*) is known for its anti-resorptive property [13]. Amalaki (*Emblica officinalis*), Ashvagandha (*Withania somnifera*), and Guduchi (*Tinospora cordifolia*) are considered prime anti-aging (Rasayana) herbs and have shown positive effects in managing osteoporosis [14-16]. Experimental studies have demonstrated that Asthiposhak Tablets have an anti-resorptive effect and promote bone re-mineralization. This is evident from the increased bone mineral density (BMD) of the femur bone and higher calcium content in the bone ash [17]. There is, however, a lack of standardized clinical evidence for the positive effects of Asthiposhak Tablets on bone health. Well-conducted clinical trials, systematic reviews, or meta-analyses would strengthen the evidence base and clarify the efficacy and safety of the tablets. Additionally, individual variability must be acknowledged, as factors like age, underlying health conditions, genetics, and lifestyle choices can influence the response to Asthiposhak Tablets, making outcomes non-uniform among individuals with Asthikshaya. Potential side effects or interactions should also be addressed to ensure the safety of users, as all therapeutic interventions, including herbal formulations, carry risks. Lastly, the lack of long-term data on the effects and potential risks of using Asthiposhak Tablets raises concerns about sustainability and necessitates further investigation.

Based on the above background, this pilot study was conducted to assess the effectiveness of Asthiposhak Tablets in individuals with Asthikshaya (osteopenia). The primary objective was to determine the efficacy of Asthiposhak Tablets in participants with osteoporosis by measuring the change in their bone mineral density (BMD) scores between visit 1 (baseline) and visit 8 (after 180 days of treatment). Secondary objectives included evaluating changes in Ayurvedic Symptom Scores, biochemical markers (serum calcium, vitamin D_3_, osteocalcin, bone-specific alkaline phosphatase levels), and assessing the safety of Asthiposhak Tablets. These parameters were measured both before (visit 2: day 0) and after the intervention (visit 8: after 180 days of treatment).

## Materials and methods

Study design and permissions

A single-center, open-label, prospective study was carried out at the Outpatient Department (OPD) 17, Department of Roga Nidana and Vikriti Vijnana, National Institute of Ayurveda, Jaipur, India. The study received approval from the Institutional Ethics Committee on May 25, 2019 (IEC/ACA/2019/1-52) and was registered with the Clinical Trials Registry of India (CTRI/2019/09/021315) on September 18, 2019. The study was conducted over a period of approximately 20-22 months, including data analysis.

Study participants

The study included consenting male and female patients between the ages of 40 and 75 years (inclusive) who had been diagnosed with Asthikshaya (Osteopenia) and had a BMD T-score ranging from -1 to -2.5. Patients who were willing to participate in the follow-up were eligible for inclusion. Additionally, patients who had a history of fractures resulting from minor injuries or falls were also considered for the study. However, patients who were taking medications known to affect bone metabolism were excluded. Patients with serum calcium levels below 2.2 mmol/L or above 2.6 mmol/L, as well as those with significant bone softening or systemic disorders during the study period, were also excluded from participation.

The study recruited participants who willingly agreed to participate and provided written informed consent. Detailed information regarding their personal, current, and past medical history was collected. A comprehensive physical examination, including vital signs, systemic assessment, and demographic data, was also conducted and documented.

Study interventions

In the single-arm study, Asthiposhak Tablets were supplied by M/s. Shree Dhootapapeshwar Limited (Batch No. DF081919, manufacturing date: May 2019 and expiry date: April 2024) and were produced in accordance with Good Manufacturing Practice (GMP) standards. The recommended dosage was two tablets to be taken three times a day with lukewarm water, for a total duration of 180 days.

Each coated Asthiposhak Tablet contains a powder formulation of the following ingredients: Kukkutandatvak Bhasma (processed hen eggshell) (100 mg), Shodhit Laksha (processed *Laccifer lacca*) (50 mg), Shodhit Guggul (processed *Commiphora wightii*) (50 mg), Choorna of Asthisamhruta (*Cissus quadrangularis*) (100 mg), Arjuna (*Terminalia arjuna*) (50 mg), Amalaki (*Emblica officinalis*) (50 mg), Ashvagandha (*Withania somnifera*) (50 mg), Guduchi (*Tinospora cordifolia*) (50 mg), and Bala (*Sida cordifolia*) (50 mg). These ingredients undergo processing in a decoction of Babboola (*Acacia arabica*) in the required quantity. The study medications, in the form of tablets, were supplied by Shree Dhootapapeshwar Limited.

Participants with additional illnesses that were not listed as exclusion criteria but required medication were still eligible for inclusion in the study, as long as there were no interactions between their current medications and the study medication. The administration of these concurrent medications was carefully documented. During each visit, the investigator inquired about any new intercurrent illnesses and the corresponding drug therapies. If necessary and if there were no interactions with the study medication, appropriate medication was prescribed and recorded for the participant.

Visit-wise schedule and assessment of efficacy and compliance

Baseline parameters, evaluation, laboratory investigations, and a bone mineral density (DEXA) scan, were conducted during visit 1, which took place between day -7 and day -3. The detailed visit-wise schedule is provided in Table 1. Participants were expected to attend their scheduled visits within the specified window period. However, participants who arrived later than the designated window period were still subjected to all trial procedures specified for that visit. Any delay in the visit was considered a protocol deviation, and appropriate corrective actions were to be taken. If necessary, the ethics committee was to be promptly informed as required.

**Table 1:** Detailed Visit-Wise Schedule of the Study Participants BMD: bone mineral density, DEXA: dual-energy X-ray absorptiometry, Hb: hemoglobin, CBC: complete blood count, ESR: erythrocyte sedimentation rate, SGOT: serum glutamic oxaloacetic transaminase (also known as AST, aspartate aminotransferase), SGPT: serum glutamic pyruvic transaminase (also known as ALT, alanine aminotransferase), Sr. Creatinine: serum creatinine, BUN: blood urea nitrogen.

No.	Activity	Visit 1 (Day -7 to Day -3) Baseline/Screening Visit	Visit 2 (Day 0 ± 7 days)	Visit 3 (Day 30 ± 7 days) and Visit 4 (Day 60 ± 7 days)	Visit 5 (Day 90 ± 7 days)	Visit 6 (Day 120 ± 7 days) and Visit 7 (Day 150 ± 7 days)	Visit 8 (Day 180 ± 7 days)
1	Informed consent process	Yes	No	No	No	No	No
2	Medical history	Yes	Yes	Yes	Yes	Yes	Yes
3	Physical examination (vital parameters and systemic examination)	Yes	Yes	Yes	Yes	Yes	Yes
4	BMD score by DEXA scan	Yes	No	No	No	No	Yes
5	Assessment of Prakriti	No	Yes	No	No	No	No
6	Assessment of Ayurvedic Symptom Score	No	Yes	Yes	Yes	Yes	Yes
7	Laboratory investigations (Hb, CBC, ESR, SGOT, SGPT, Sr. Creatinine, and BUN)	Yes	No	No	No	No	Yes
8	Laboratory investigations (serum calcium, vitamin D_3_, bone-specific alkaline phosphatase, and osteocalcin levels)	No	Yes	No	No	No	Yes
9	Adverse events	Yes	Yes	Yes	Yes	Yes	Yes

The effectiveness of the treatment was evaluated by assessing the T-score, Ayurvedic Symptom Score, and levels of biochemical markers. The T-score was determined through DEXA scans to estimate bone mineral density. A T-score of -1.0 or above indicated normal bone density, a T-score between -1.0 and -2.5 indicated low bone density or osteopenia, and a T-score of -2.5 or below indicated osteoporosis. The Ayurvedic Symptom Score table can be found in Table 2. Biochemical markers such as bone-specific alkaline phosphatase (ALP), osteocalcin (OC), serum calcium, and vitamin D3 were measured. Significant statistical changes in these parameters were evaluated between visit 2 (day 0) and visit 8 (after 180 days of treatment). Safety assessments included monitoring adverse events (AEs), vital signs (temperature, pulse rate, respiratory rate, and blood pressure), systemic examination, and blood investigations such as complete blood count, liver function test, and renal function test, as outlined in the visit-wise schedule provided in Table 1. Drug interactions were assessed by analyzing patient-reported adverse effects or the absence of desired medication effectiveness. In the case of participant withdrawals or dropouts, the investigator conducted clinical assessments and noted the reasons for discontinuation from the study, which were recorded in the Case Report Form (CRF).

**Table 2:** Ayurvedic Symptom Score

Serial Number	Efficacy Variable	Interpretation	Scores/Grades
1	Asthishoola (Asthibheda) (Pain in bones):	No piercing pain in bones	0
		Mild piercing pain in bones not affecting daily activities	1
		Occasional moderate piercing pain in bones not affecting daily activities. (Pain can be relieved during daily activities with no need for medication.)	2
		Frequently severe piercing pain in bones affecting daily activities. Patients need medication.	3
		Continuous severe piercing pain in bones with restricted movements not relieved by even simple medication.	4
	Visual Analogue Scale: To assess the severity of pain.		
2	Vaivarnata (Skin discoloration):	Present	0
		Absent	1
3	Kesha Vikara (Palitya) (Graying of hair):	No graying of hair	0
		Very few gray hair	1
		Partial graying of hair	2
		Significant graying of hair	3
		Generalized graying of hair	4
4	Keshapata (Hairfall):	No hairfall	0
		Hairfall once in the morning while washing/combing	1
		Hairfall on every time of combing	2
		Hairfall even without combing and raised hairline in frontal region (mild baldness)	3
		Visible or significant baldness in frontal or vertex region.	4
5	Shrama (Tiredness):	No tiredness	0
		Tiredness with excessive exertion	1
		Tiredness with moderate exertion	2
		Tiredness with mild exertion	3
		Tiredness with no exertion	4
6	Sandhi Shaithilya (Looseness/weakness in joints):	No feeling of looseness/weakness in joints	0
		Mild feeling of looseness/weakness in joints. Patient can stand/walk independently without difficulty.	1
		Moderate feeling of looseness/weakness in joints. Patient can stand/walk independently with difficulty.	2
		Severe feeling of looseness/weakness in joints. Patient can stand/walk only with support. (crutches, cane, walkers)	3
		Severe feeling of looseness/weakness in joints. Patient unable to stand/walk even with support (crutches, cane, walkers).	4
7	Nakha Vikara (Nail deformity):	No nail deformity	0
		Mild loss of natural texture and malleability of nails	1
		Moderate loss of natural texture and malleability of nails	2
		Visible brittleness of nails which breaks easily	3
8	Raukshya (Dryness):	No dryness	0
		Occasional dryness without winter season. Visible dryness, mild dull white streaks after scratching on the skin which disappear after some time.	1
		Visible dryness, mild dull white streaks after scratching on the skin which disappear after some time.	2
		Dryness/roughness, bright white streaks on the skin remaining for a considerable time.	3
		Dryness/roughness and criss-cross visible cracking of skin.	4
9	Mansa Abhilasha (Desire of eating meat):	Absent	0
		Present, occasional	1
		Present, intense	2
10	Asthi-sandhi Baddhata (Joint stiffness):	No stiffness	0
		Stiffness for few minutes, relieved by mild movements.	1
		Stiffness lasting for 1 to 2 hours, but routine work are not disturbed.	2
		Stiffness lasting for more than 2 hours mildly affecting the daily routine.	3
		Episode of stiffness lasting for 2-6 hours. Daily routine are hampered.	4
11	Katishoola (Backache):	No backache	0
		Occasionally	1
		Relieved by medicine	2
		Dependent on painkiller	3
12	Sandhishoola (Pain in joints):	No pain	0
		Mild pain and difficulty in walking	1
		Slight difficulty in walking	2
		Much difficulty in walking	3
13	Parvabheda (Pain in finger joints):	No pain in phalanges	0
		Mild pain in phalanges	1
		Discomforting pain in phalanges	2
		Distressing pain in phalanges	3
		Horrible pain in phalanges	4
	Visual Analogue Scale: To assess the severity of pain.		

The study medication was provided to participants during visit 2, visit 3, visit 4, visit 5, visit 6, and visit 7. During visit 2 (day 0), participants received a container containing the study medication, which included 222 Asthiposhak Tablets, to be taken over a period of 37 days (30 ± 7 days). Compliance with the medication was assessed by calculating the number of unused tablets at each subsequent visit, starting from visit 3 (day 30 ± 7) up to visit 8 (day 180 ± 7), by the study coordinator. Compliance was determined using the following formula: percentage compliance = (number of tablets taken/number of tablets given) x 100. Compliance within the range of 80% to 125% was considered normal compliance.

Sample size and statistical analysis

As this study was a pilot and the first conducted study in humans, no formal sample size calculation was performed. A sample size of 30 study participants was chosen. The efficacy and safety data were analyzed using the 'intention-to-treat' analysis approach with SPSS version 21 software (IBM Corporation, Armonk, New York, United States). The normality of the data was assessed using the Wilk-Shapiro test, and descriptive statistics were used for analysis. The data were presented as percentages, mean ± SD, or median (IQR).

To compare data at different intervals, paired t-tests or Wilcoxon signed-rank tests were used. One-way analysis of variance (ANOVA) with Bonferroni correction was employed to determine any significant differences in Ayurvedic Symptom Score between the visits. Friedman's two-way analysis of variance by ranks was used to assess any variations in vital parameters. The level of significance used in the study was set at p<0.05.

## Results

**Figure 1:**
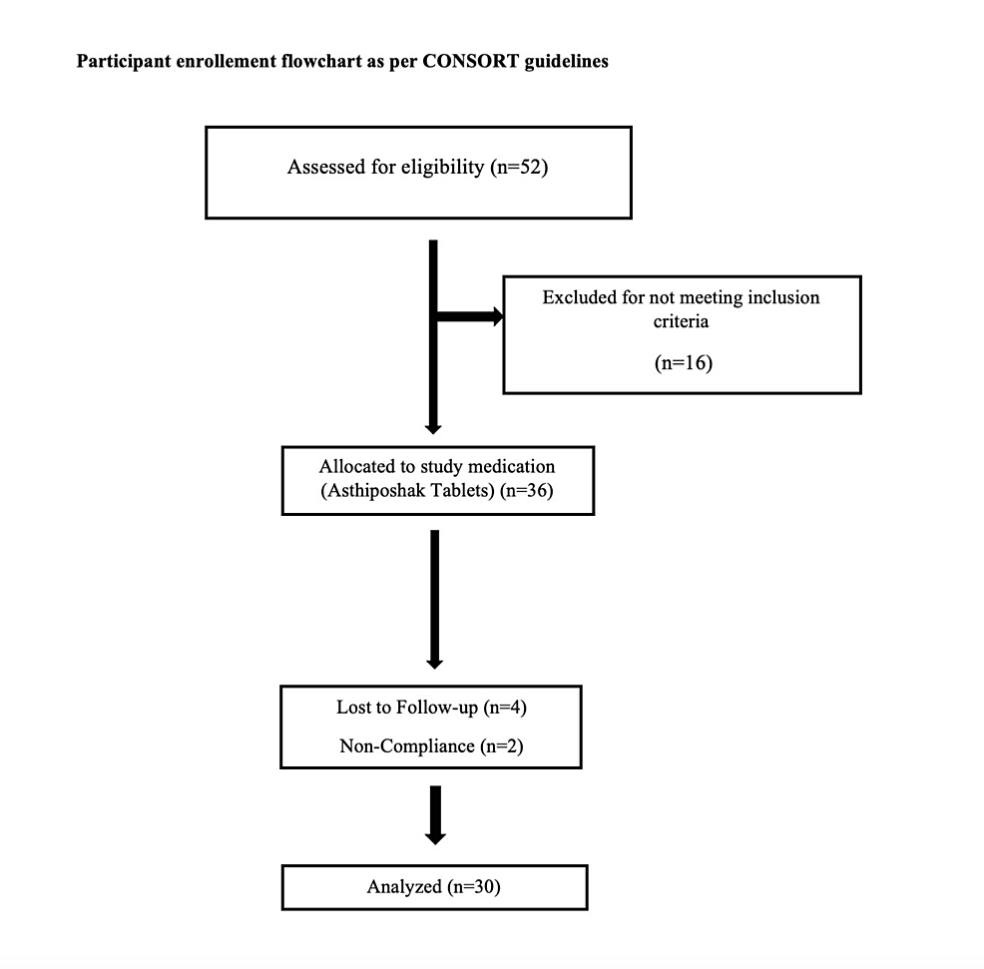
CONSORT Flow Diagram for the Enrolment of the Participants CONSORT: Consolidated Standards of Reporting Trials.

A total of 52 participants were screened for eligibility to participate in the study following written informed consent (Figure 1). Of these, 16 participants were not found to be eligible. Thus, 36 participants were recruited for the study and were given the study medication, i.e., Asthiposhak Tablets as per the protocol. Of these, 30 participants completed the study. Six participants dropped out from the study, of which four were due to loss to follow-up (two participants lost to follow-up on visit 3 and two participants lost to follow-up on visit 4) and two participants were withdrawn on visit 5 due to poor compliance. Demographic and Prakriti details of the participants are described in Table 3. The mean age of participants in the study was 51.33 ± 8.31 years, and 60% (18) of the participants were females.

**Table 3:** Effect of Asthiposhak on the Ayurvedic Symptom Score

Variables	Visit 2 (V2) (Day 0)	Visit 5 (V5) (Day 90 ± 7)	Visit 8 (V8) (Day 180 ± 7)	p-value (V5 vs. V2)	p-value (V8 vs. V2)	p-value (V8 vs. V5)
Mean Total Ayurvedic Symptom Score	20.93 ± 7.83	12.10 ± 5.53	7.10 ± 4.68	p<0.01	p<0.01	p<0.01
Ayurvedic Symptom Score of Asthishoola	2.57 ± 1.14	1.67 ± 0.84	0.70 ± 0.65	p<0.05	p<0.05	p<0.05
Ayurvedic Symptom Score of Keshapata	1.47 ± 0.97	0.60 ± 0.86	0.33 ± 0.84	p<0.05	p<0.05	p>0.05
Ayurvedic Symptom Score of Shrama	2.50 ± 1.00	1.33 ± 0.84	0.53 ± 0.57	p<0.05	p<0.05	p<0.05
Ayurvedic Symptom Score of Sandhi Shaithilya	1.97 ± 0.72	1.10 ± 0.80	0.63 ± 0.72	p<0.05	p<0.05	p>0.05
Ayurvedic Symptom Score of Nakha Vikara	1.13 ± 0.16	0.73 ± 0.11	0.50 ± 0.11	p>0.05	p<0.05	p>0.05
Ayurvedic Symptom Score of Raukshya	1.70 ± 1.12	0.73 ± 0.83	0.60 ± 0.50	p<0.05	p<0.05	p>0.05
Ayurvedic Symptom Score of Asthi-sandhi Baddhata	2.60 ± 0.97	1.40 ± 0.93	0.87 ± 0.78	p<0.05	p<0.05	p>0.05
Ayurvedic Symptom Score of Katishoola	1.97 ± 0.77	1.17 ± 0.79	0.27 ± 0.58	p<0.05	p<0.05	p<0.05
Ayurvedic Symptom Score of Sandhishoola	1.77 ± 0.81	0.93 ± 0.87	0.37 ± 0.62	p<0.05	p<0.05	p<0.05
Ayurvedic Symptom Score of Parvabheda	1.27 ± 0.98	0.57 ± 0.63	0.37 ± 0.49	p<0.05	p<0.05	p>0.05

Effect of Asthiposhak Tablets on bone mineral density (BMD) (T-score)

**Figure 2:**
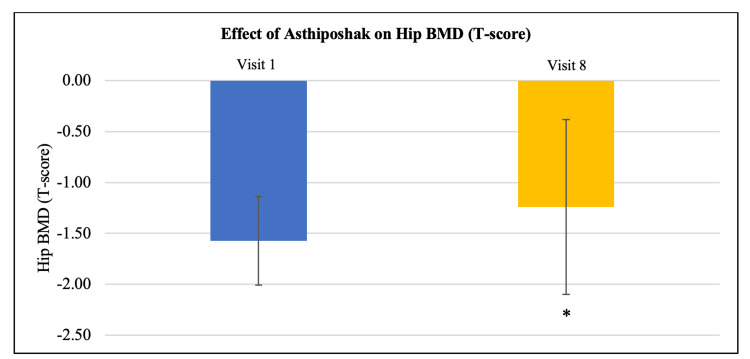
Effect of Asthiposhak Tablets on Hip Bone Mineral Density (BMD) (T-Score) p<0.05 was considered significant. Values expressed as mean ± SD. *p<0.01 vs. visit 1 (baseline). Paired t-test/Wilcoxon signed-rank test was employed as per the normality testing of data.

**Figure 3:**
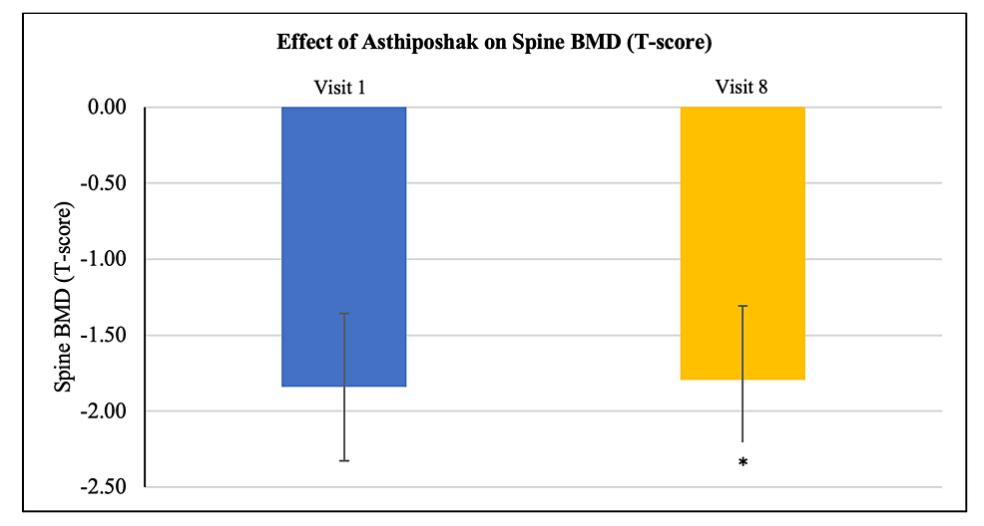
Effect of Asthiposhak Tablets on Spine Bone Mineral Density (BMD) (T-Score) p<0.05 was considered significant. Values expressed as mean ± SD. *p<0.01 vs. visit 1 (baseline). Paired t-test/Wilcoxon signed-rank test was employed as per the normality testing of data.

**Figure 4:**
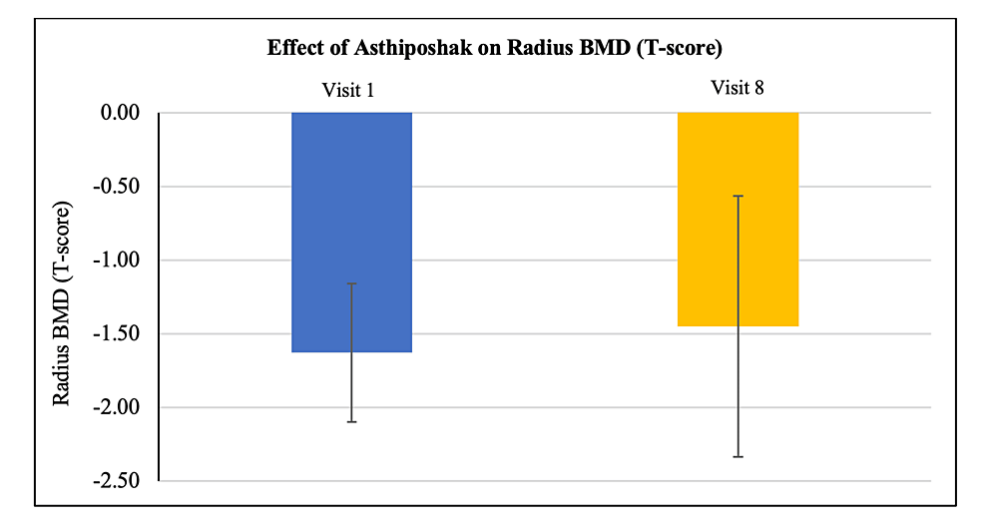
Effect of Asthiposhak Tablets on Radius Bone Mineral Density (BMD) (T-Score) p<0.05 was considered significant. Values expressed as mean ± SD. p>0.01 vs. visit 1 (baseline). Paired t-test/Wilcoxon signed-rank test was employed as per the normality testing of data.

Out of 30 participants, BMD (T-score) suggestive of osteopenia (-1 to -2.5) at the hip, spine, and radius was seen in 22 participants, 21 participants, and 14 participants, respectively. Improvement in mean BMD (T-score) was seen at visit 8 (after 180 days of treatment), as compared to the BMD (T-score) at visit 1 (baseline) in the 30 participants who completed the study. Significant improvement was seen in the mean BMD (T-score) of hip and spine regions (p<0.01). Although the mean BMD (T-score) of radius also improved, it was not statistically significant. Improvement in BMD (T-score) after treatment with Asthiposhak Tablets indicates its bone-strengthening effect (Figures 2-4).

Effect of Asthiposhak Tablets on the Total Ayurvedic Symptom Score

**Figure 5:**
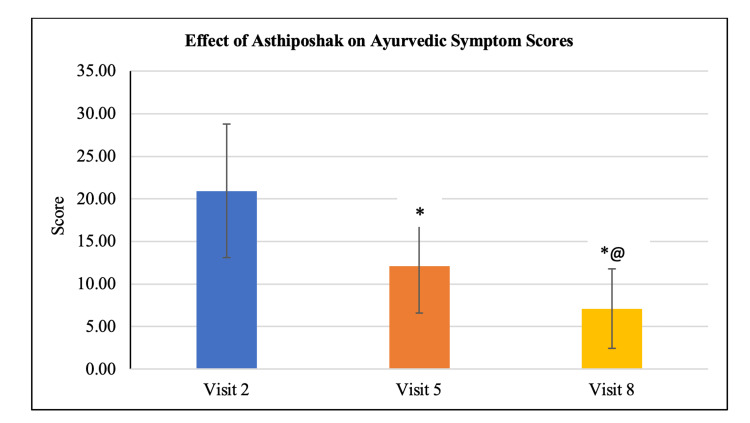
Effect of Asthiposhak on the Total Ayurvedic Symptom Score p<0.05 was considered significant. Values are expressed as mean ± SD. *p<0.01 vs. visit 2 (day 0), @p<0.01 vs. visit 5 (day 90). Analysis of variance (ANOVA) with Bonferroni correction was employed to test the significance between the visits.

The mean Total Ayurvedic Symptom Score at visit 2 (day 0) was 20.93 ± 7.83, which significantly (p<0.01) reduced to 12.10 ± 5.53 and 7.10 ± 4.68 at visit 5 (after 90 days of treatment) and visit 8 (after 180 days of treatment - final Visit), respectively. Also, the reduction in the mean Total Ayurvedic Symptom Score at visit 8 was significant (p<0.01) as compared to the score at visit 5. The reduction in Total Ayurvedic Symptom Score along with improvement in BMD (T-score) indicates the positive role of Asthiposhak Tablets in improving bone health and associated symptoms in cases of Asthikshaya (osteopenia) (Table 3 and Figure 5).

**Figure 6:**
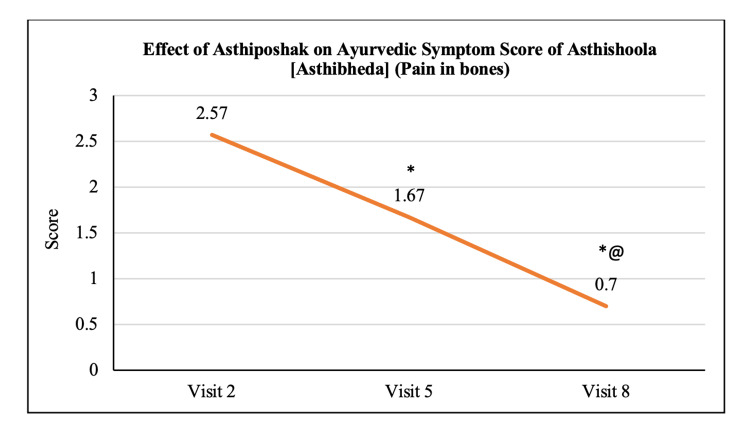
Effect of Asthiposhak on Ayurvedic Symptom Score of Asthishoola (Asthibheda) (Pain in Bones) p<0.05 was considered significant. Values are expressed as mean ± SD. *p<0.05 vs. visit 2 (day 0), @p<0.05 vs. visit 5. Analysis of variance (ANOVA) with Bonferroni correction was employed to test the significance between the visits.

**Figure 7:**
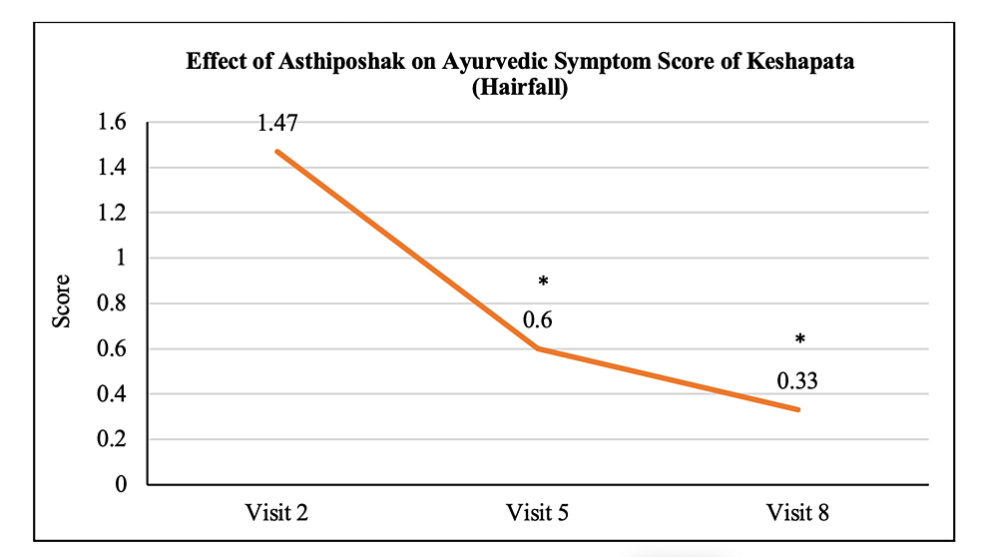
Effect of Asthiposhak on Ayurvedic Symptom Score of Keshapata (Hairfall) p<0.05 was considered significant. Values are expressed as mean ± SD. *p<0.05 vs. visit 2 (day 0). Analysis of variance (ANOVA) with Bonferroni correction was employed to test the significance between the visits.

**Figure 8:**
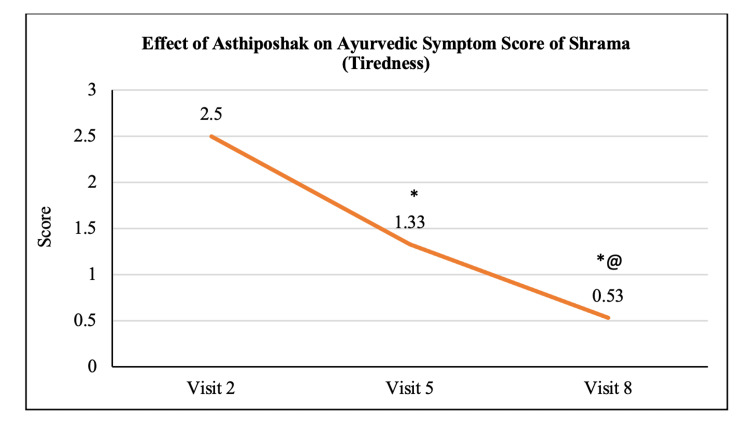
Effect of Asthiposhak on Ayurvedic Symptom Score of Shrama (Tiredness) p<0.05 was considered significant. Values are expressed as mean ± SD. *p<0.05 vs. visit 2 (day 0), @p<0.05 vs. visit 5 (day 90). Analysis of variance (ANOVA) with Bonferroni correction was employed to test the significance between the visits.

**Figure 9:**
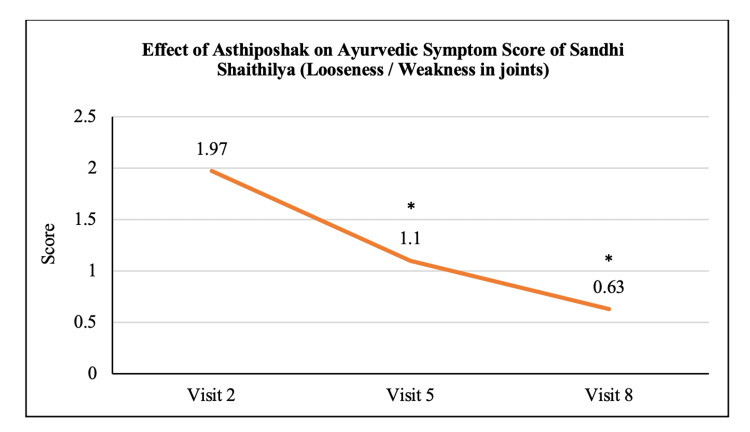
Effect of Asthiposhak on Ayurvedic Symptom Score of Sandhi Shaithilya (Looseness/Weakness in Joints) p<0.05 was considered significant. Values are expressed as mean ± SD. *p<0.05 vs. visit 2 (day 0). Analysis of variance (ANOVA) with Bonferroni correction was employed to test the significance between the visits.

The mean score of Asthishoola (pain in bones) at visit 2 (day 0) was 2.57 ± 1.14 which significantly reduced to 1.67 ± 0.84 and 0.70 ± 0.65 at visit 5 (after 90 days of treatment) and visit 8 (after 180 days of treatment - final visit), respectively. Also, the reduction in the mean Asthishoola score at visit 8 was significant (p<0.05) as compared to the score at visit 5 (Figure 6). The mean score of Keshapata (hairfall) at visit 2 (day 0) was 1.47 ± 0.97 which significantly (p<0.05) reduced to 0.60 ± 0.86 and 0.33 ± 0.84 at visit 5 (after 90 days of treatment) and visit 8 (after 180 days of treatment - final visit), respectively. The reduction in Keshapata score at visit 8 was not significant as compared to visit 5 (Figure 7). The mean score of Shrama (tiredness) at visit 2 (day 0) was 2.50 ± 1.00 which significantly (p<0.05) reduced to 1.33 ± 0.84 and 0.53 ± 0.57 at visit 5 (after 90 days of treatment) and visit 8 (after 180 days of treatment - final visit), respectively. Also, the reduction in Shrama score at visit 8 was significant as compared to visit 5 (Figure 8). The mean score of Sandhi Shaithilya (looseness/weakness in joints) at visit 2 (day 0) was 1.97 ± 0.72 which significantly (p<0.05) reduced to 1.10 ± 0.80 and 0.63 ± 0.72 at visit 5 (after 90 days of treatment) and visit 8 (after 180 days of treatment - final visit), respectively. The reduction in Sandhi Shaithilya score at visit 8 was not significant as compared to visit 5 (Figure 9).

**Figure 10:**
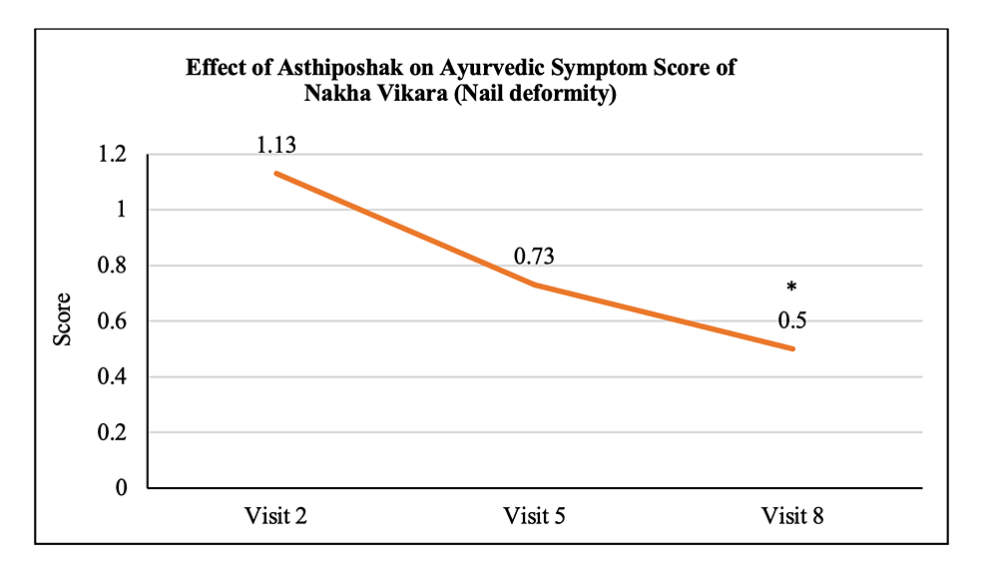
Effect of Asthiposhak on Ayurvedic Symptom Score of Nakha Vikara (Nail Deformity) p<0.05 was considered significant. Values are expressed as mean ± SD. *p<0.05 vs. visit 2 (day 0). Analysis of variance (ANOVA) with Bonferroni correction was employed to test the significance between the visits.

**Figure 11:**
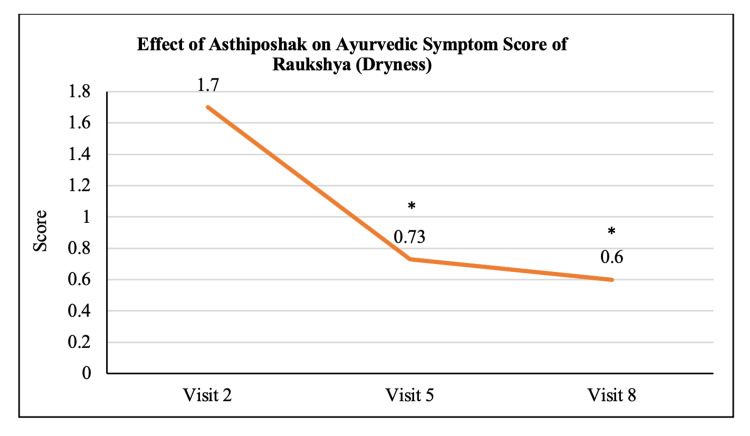
Effect of Asthiposhak on Ayurvedic Symptom Score of Raukshya (Dryness) p<0.05 was considered significant. Values are expressed as mean ± SD. *p<0.05 vs. visit 2 (day 0). Analysis of variance (ANOVA) with Bonferroni correction was employed to test the significance between the visits.

**Figure 12:**
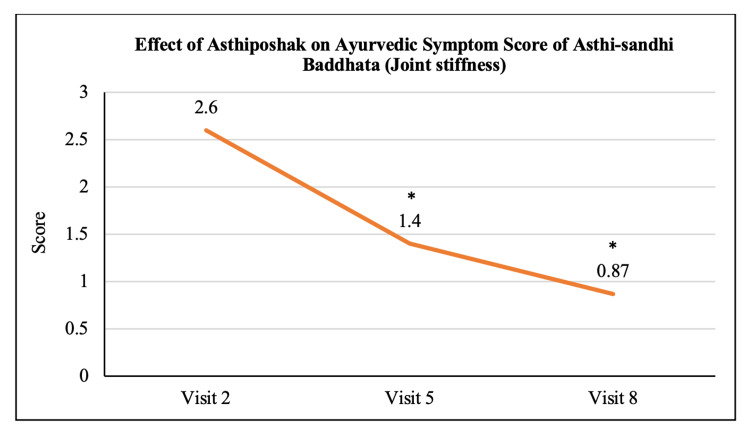
Effect of Asthiposhak on Ayurvedic Symptom Score of Asthi-sandhi Baddhata (Joint Stiffness) p<0.05 was considered significant. Values are expressed as mean ± SD. *p<0.05 vs. visit 2 (day 0). Analysis of variance (ANOVA) with Bonferroni correction was employed to test the significance between the visits.

**Figure 13:**
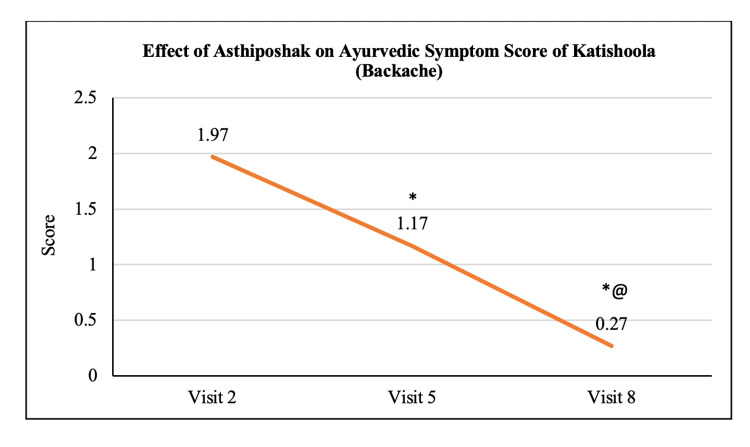
Effect of Asthiposhak on Ayurvedic Symptom Score of Katishoola (Backache) p<0.05 was considered significant. Values are expressed as mean ± SD. *p<0.05 vs. visit 2 (day 0), @p<0.05 vs. visit 5 (day 90). Analysis of variance (ANOVA) with Bonferroni correction was employed to test the significance between the visits.

**Figure 14:**
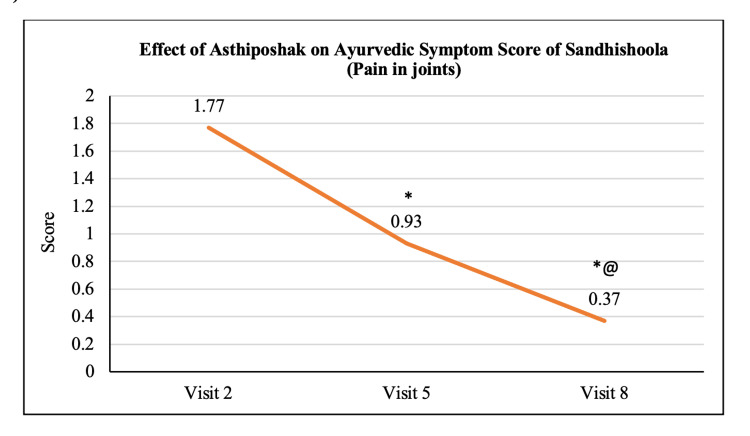
Effect of Asthiposhak on Ayurvedic Symptom Score of Sandhishoola (Pain in Joints) p<0.05 was considered significant. Values are expressed as mean ± SD. *p<0.05 vs. visit 2 (day 0), @p<0.05 vs. visit 5 (day 90). Analysis of variance (ANOVA) with Bonferroni correction was employed to test the significance between the visits.

**Figure 15:**
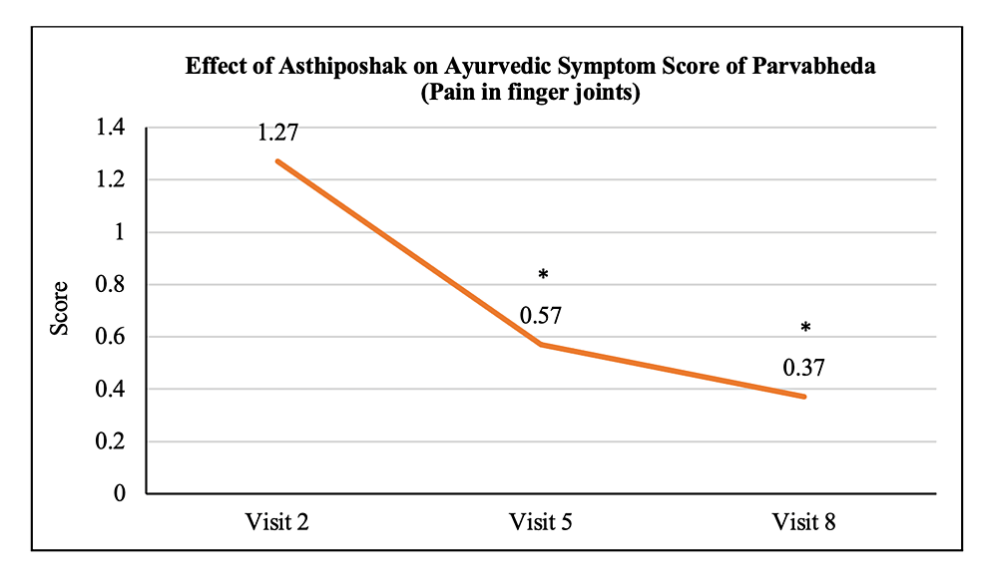
Effect of Asthiposhak on Ayurvedic Symptom Score of Parvabheda (Pain in Finger Joints) p<0.05 was considered significant. Values are expressed as mean ± SD. *p<0.05 vs. visit 2 (day 0). Analysis of variance (ANOVA) with Bonferroni correction was employed to test the significance between the visits.

The mean score of Nakha Vikara (nail deformity) at visit 2 (day 0) was 1.13 ± 0.90 which reduced to 0.73 ± 0.64 and 0.50 ± 0.63 at visit 5 (after 90 days of treatment) and visit 8 (after 180 days of treatment - final visit), respectively. The reduction at visit 8 was statistically (p<0.05) significant as compared to visit 2. The reduction in Nakha Vikara Score at visit 5 was not significant to visit 2 and also the score at visit 8 was not significant to visit 5 (Figure 10). The mean score of Raukshya (dryness) at visit 2 (day 0) was 1.70 ± 1.12 which significantly (p<0.05) reduced to 0.73 ± 0.83 and 0.60 ± 0.50 at visit 5 (after 90 days of treatment) and visit 8 (after 180 days of treatment - final visit), respectively. The reduction in Raukshya score at visit 8 was not significant as compared to visit 5 (Figure 11). The mean score of Asthi-sandhi Baddhata (joint stiffness) at visit 2 (day 0) was 2.60 ± 0.97 which significantly (p<0.05) reduced to 1.40 ± 0.93 and 0.87 ± 0.78 at visit 5 (after 90 days of treatment) and visit 8 (after 180 days of treatment - final visit), respectively. The reduction in Asthi-sandhi Baddhata score at visit 8 was not significant as compared to visit 5 (Figure 12). The mean score of Katishoola (Backache) at visit 2 (day 0) was 1.97 ± 0.77, which significantly reduced to 1.17 ± 0.79 and 0.27 ± 0.58 at visit 5 (after 90 days of treatment) and visit 8 (after 180 days of treatment - final visit), respectively. Also, the reduction in Katishoola score at visit 8 was significant (p<0.05) as compared to visit 5 (Figure 13). The mean score of Sandhishoola (pain in joints) at visit 2 (day 0) was 1.77 ± 0.81, which significantly (p<0.05) reduced to 0.93 ± 0.87 and 0.37 ± 0.62 at visit 5 (after 90 days of treatment) and visit 8 (after 180 days of treatment - final visit), respectively. Also, the reduction in Sandhishoola score at visit 8 was significant (p<0.05) as compared to visit 5 (Figure 14). The mean score of Parvabheda (pain in finger joints) at visit 2 (day 0) was 1.27 ± 0.98, which significantly (p<0.05) reduced to 0.57 ± 0.63 and 0.37 ± 0.49 at visit 5 (after 90 days of treatment) and visit 8 (after 180 days of treatment - final visit), respectively. The reduction in Parvabheda score at visit 8 was not significant as compared to visit 5 (Figure 15).

Effect of Asthiposhak Tablets on the levels of biochemical markers

**Figure 16:**
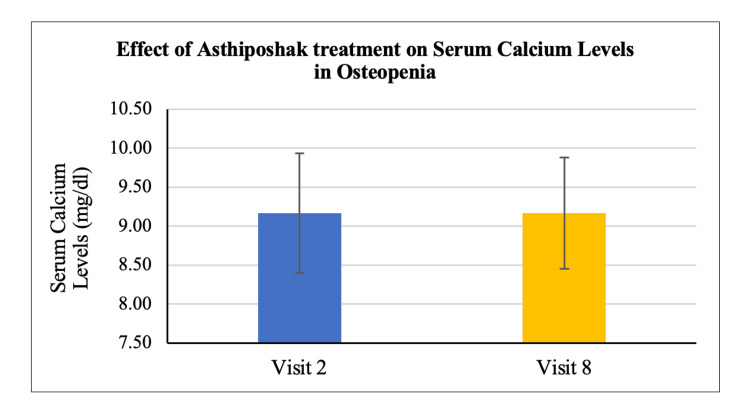
Effect of Asthiposhak Treatment on Serum Calcium Levels in Osteopenia p<0.05 was considered significant. Results are expressed as mean ± SD. Paired t-test was used.

**Figure 17:**
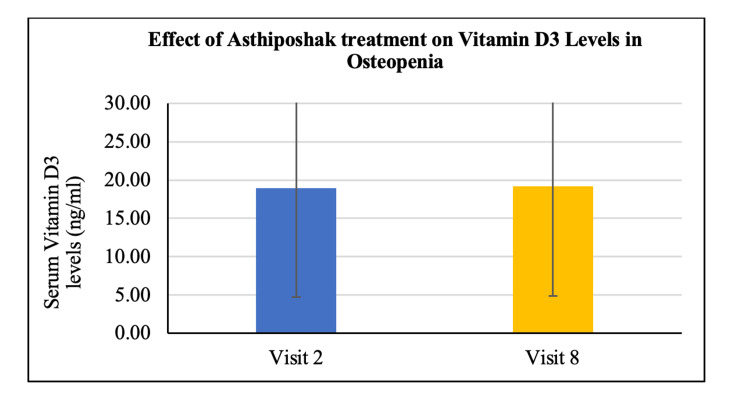
Effect of Asthiposhak Treatment on Vitamin D3 Levels in Osteopenia p<0.05 was considered significant. Results are expressed as mean ± SD. Wilcoxon signed-rank test was used.

**Figure 18:**
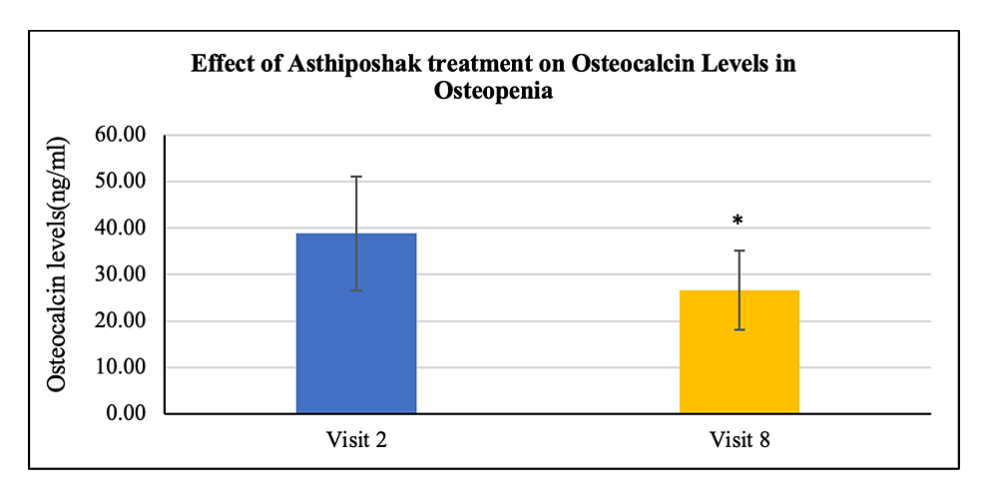
Effect of Asthiposhak Treatment on Osteocalcin Levels in Osteopenia p<0.05 was considered significant. Results are expressed as mean ± SD. *p<0.01 vs. visit 2 (day 0). Wilcoxon signed-rank test was used.

**Figure 19:**
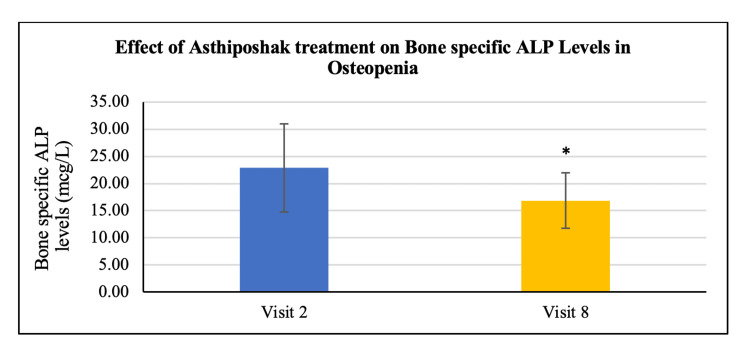
Effect of Asthiposhak treatment on Bone-Specific Alkaline Phosphatase (ALP) Levels in Osteopenia p<0.05 was considered significant. Results are expressed as mean ± SD. *p<0.01 vs. visit 2 (day 0). Wilcoxon signed-rank test was used.

No statistically significant change/improvement was seen in the levels of serum calcium and serum vitamin D_3_ at visit 8 (after 180 days of treatment) as compared to visit 2 (day 0) (Figures 16, 17). A statistically significant change/reduction was seen in the levels of serum osteocalcin and bone-specific alkaline phosphatase (ALP) at visit 8 (after 180 days of treatment - final visit) as compared to visit 2 (day 0) (Figures 18, 19).

Safety parameters

No adverse events or drug interactions were reported/observed at all time points of measurement, i.e., visit 2 (day 0), visit 3 (day 30 ± 7), visit 4 (day 60 ± 7), visit 5 (day 90 ± 7), visit 6 (day 120 ± 7), visit 7 (day 150 ± 7), and visit 8 (day 180 ± 7). No further evaluation was carried out for long-term safety data. All vital parameters, i.e., temperature, blood pressure, heart rate, and respiratory rate, were found to be within normal ranges, at all time points of measurement. No statistically significant change was observed in any vital parameter of the 30 participants, measured on visit 2 (day 0), visit 3 (day 30 ± 7), visit 4 (day 60 ± 7), visit 5 (day 90 ± 7), visit 6 (day 120 ± 7), visit 7 (day 150 ± 7), and visit 8 (day 180 ± 7).

## Discussion

Despite the availability of various pharmacological therapies for osteoporosis, the prevalence of the disease in India continues to rise. This can be attributed to factors such as underdiagnosis of osteopenia and osteoporosis, low patient adherence to pharmacological therapy due to side effects, and the high cost of treatment [5,18]. In such circumstances, preventive treatment using traditional systems of medicine may provide a ray of hope for patients, as it can help reduce the risk of fractures [19]. To investigate the effectiveness of Asthiposhak Tablets, a proprietary formulation available in the Indian market, in managing osteopenia (reduced bone density), a clinical study was conducted.

Thirty-six eligible participants were enrolled in the study and received Asthiposhak Tablets according to the prescribed protocol. Out of these, 30 participants successfully completed the study. Six participants discontinued their participation, with four being lost to follow-up and two withdrawn due to poor compliance.

The primary objective of the study was to evaluate the changes in BMD T-scores after 180 days of treatment compared to the initial baseline measurements. The T-scores were calculated and recorded at three different sites: hip, spine, and radius. Out of the 30 participants, 22 participants had BMD T-scores indicating osteopenia in the hip, 21 participants in the spine, and 14 participants in the radius. Significant improvements in BMD T-scores were observed in the hip and spine regions. However, although there was the improvement in BMD T-scores at the radius, it did not reach statistical significance. A similar study conducted by Munshi et al. [20] examined the effectiveness of Panchatikta Ghrita as an additional therapy to vitamin D_3_ and calcium supplements in treating osteopenia. In that study, the improvement in BMD T-scores was not statistically significant [20].

The secondary objectives of the study involved evaluating various biochemical bone markers in the serum, including vitamin D_3_, calcium, osteocalcin, and bone-specific alkaline phosphatase (ALP) levels. There were no significant changes or differences observed in serum vitamin D3 and calcium levels between visit 2 and visit 8. The maintenance of these levels without deterioration may indicate control of bone turnover [21]. However, a significant reduction was observed in serum osteocalcin and bone-specific ALP levels after 180 days of treatment with Asthiposhak Tablets. Elevated levels of osteocalcin are associated with increased osteoblast activity, indicating higher bone turnover. ALP is commonly used in clinical settings. The significant reduction in serum osteocalcin and bone-specific ALP levels at visit 8, compared to the levels at visit 2, suggests a decrease in the control of bone turnover. The improvement in bone turnover markers observed in this study was higher compared to the study conducted by Munshi et al. [20].

In addition to evaluating BMD and bone turnover markers, the effectiveness of Asthiposhak Tablets was assessed using the Ayurvedic Symptom Score. This score was developed based on Ayurvedic scriptures, publications, and guidelines that identify the signs and symptoms of Asthikshaya (reduced bone density) and Asthi Pradoshaja Vikara (disorders related to bones). A notable reduction was observed in the mean Total Ayurvedic Symptom Score at visit 5 and visit 8 compared to the score recorded at visit 2. Furthermore, the reduction at visit 8 was found to be statistically significant when compared to visit 5.

Regarding the specific domains of the Ayurvedic Symptom Score, significant reductions were observed at visit 5 and visit 8 compared to visit 2 in several areas. These included Asthishoola (bone pain), Keshapata (hairfall), Shrama (tiredness), Sandhi Shaithilya (joint looseness/weakness), Raukshya (dryness), Asthi-sandhi Baddhata (joint stiffness), Katishoola (backache), Sandhishoola (joint pain), and Parvabheda (finger joint pain). Additionally, the domain Nakha Vikara (nail deformity) showed a significant improvement only at visit 8 when compared to visit 2.

There were no instances of serious adverse events (SAEs) or drug interactions throughout the entire duration of the study. Systemic examination parameters were consistently found to be within normal limits at all measurement time points. Safety variables, including bone turnover markers, were within the normal range at both visit 1 and visit 8. However, due to the impact of coronavirus disease 2019 (COVID-19), the estimation of bone turnover markers at visit 5 was possible for only 15 out of the 30 participants. Additionally, safety parameters were assessed solely at visit 1 and visit 8.

The study has certain limitations based on its methodology. Firstly, the sample size was relatively small (pilot sample), with only 30 participants. To improve reliability, future studies should consider recruiting a larger and more diverse sample. Secondly, the study was conducted at a single center, which limits the representation of different populations. Conducting multi-center studies across various locations would enhance the external validity of the results. Another limitation is the open-label design which could introduce biases and affect result interpretation. Utilizing a double-blind, randomized controlled trial design in future studies would help mitigate these limitations. Additionally, due to the impact of the COVID-19 pandemic, bone turnover markers were assessed in a limited number of participants at visit 5, and safety parameters were only evaluated at visit 1 and visit 8. More comprehensive assessments of safety parameters and bone turnover markers throughout the study duration would provide a more comprehensive understanding of the long-term effects and safety profile of Asthiposhak Tablets.

## Conclusions

In conclusion, the study investigated the effectiveness of Asthiposhak Tablets in managing osteopenia and yielded significant findings. Improvements in BMD T-scores were observed in the hip and spine regions, indicating a positive effect on bone density. Significant reductions in serum osteocalcin and bone-specific ALP levels indicated a decrease in bone turnover. The Ayurvedic Symptom Score demonstrated notable improvements in various domains, including bone pain, joint stiffness, and fatigue. No serious adverse events were reported, and safety parameters remained within normal limits. However, the study has limitations, such as a small sample size, single-center and single-arm design, open-label approach, and limited assessments of bone turnover markers and safety parameters due to COVID-19 limitations. Future studies examining the potential of Asthiposhak Tablets for managing osteopenia should encompass larger sample sizes, multi-center settings, double-blind randomized controlled designs, and comprehensive evaluations of long-term effects and safety. Additionally, to enhance the study population's homogeneity, it is advisable to consider factors such as age, gender, inclusion of control and placebo groups, blinding, and matching participants based on body mass index (BMI). Overall, the study's findings contribute to the broader context of osteopenia and osteoporosis management, highlighting the potential benefits of herbal and traditional medicine interventions such as Asthiposhak Tablets in this field.
